# What makes community‐based physical activity programs for culturally and linguistically diverse older adults effective? A systematic review

**DOI:** 10.1111/ajag.12815

**Published:** 2020-06-29

**Authors:** Jed Montayre, Stephen Neville, Ihaka Dunn, Jagamaya Shrestha‐Ranjit, Valerie Wright‐St. Clair

**Affiliations:** ^1^ School of Nursing and Midwifery Western Sydney University Penrith NSW Australia; ^2^ Ageing and Wellbeing and Head of Nursing Department School of Clinical Sciences Auckland University of Technology Auckland New Zealand; ^3^ School of Clinical Sciences Auckland University of Technology Auckland New Zealand

**Keywords:** CALD, culturally appropriate interventions, exercise program, integrative review

## Abstract

**Objective:**

This integrative review aimed to determine the features of effective physical activity programs for culturally and linguistically diverse (CALD) older adults.

**Methods:**

We searched for relevant articles in MEDLINE, PubMed, Google Scholar, Scopus and CINAHL. Articles were selected for evaluation if they included CALD older adults and implemented physical activity programs with culturally specific design features. Consistent with the Whittemore and Knafl integrative review methodology, we used the Downs and Black Checklist, Mixed Methods Appraisal Tool and the McMaster University appraisal tool for quantitative studies to assess the quality of included articles.

**Results:**

Seven articles were included in this review. Effective community‐based exercise or physical activity programs for CALD populations commonly featured close‐to‐home delivery, native language instruction and adaptations of culturally familiar activities.

**Conclusion:**

The provision of culturally appropriate community‐based physical activity programs appears to support and encourage engagement among CALD older adults.

**Policy Impact**

Protocols and guidelines designed to encourage and increase CALD older adults’ participation in physical activities should ensure that culturally familiar activities are provided at a convenient and accessible location and in the person's own language.

**Practice Impact**

Essential aspects for ensuring successful participation by CALD older adults in physical activity programs include providing the program at a convenient and accessible location and in an older person's native language, and ensuring activities are culturally familiar.

## 1 INTRODUCTION

There is strong evidence to suggest regular physical activity positively impacts older adults’ health and well‐being. Regular physical exercise in older adult populations has been shown to decrease the risk for falls, osteoporotic changes and muscular atrophy, improve sleep and blood pressure, and enhance mood and general well‐being.^1^ Exercise is also known to reduce functional disability,^2^, ^3^ delay cognitive decline,^4^ improve mental health^5^ and promote social relationships.^6^ In addition to engaging in structured exercise, healthy levels of physical activity can be achieved through various domestic family‐ and community‐based activities. These activities include undertaking everyday chores, physical leisure activities (eg gardening or dancing) and walking or cycling as modes of transport.^7^ From a global perspective, the World Health Organization (WHO)^7^ recommends target physical activity levels for older adults are ‘irrespective of gender, race, ethnicity or income level’.^7^ However, the WHO acknowledged that the communication and implementation strategies in physical activity programs must be tailored for diverse and often disparate groups within populations.^7^ At a country level, physical activity recommendations in New Zealand^8^ advise the use of culturally specific activities tailored for particular cultural or ethnic groups.^8^ Examples of programs that used culture‐specific design principles include a cardiorespiratory fitness study involving Pacific adults^9^ that focused on engagement with Pacific church leaders and communities. Further, Rolleston, Doughty and Poppe^10^ implemented a co‐designed Kaupapa Māori clinical exercise and lifestyle management program using a culture‐specific approach. Despite the availability of global and country‐specific physical activity guidelines and frameworks, physical inactivity in older adults contributes to the worldwide health burden. A systematic review focusing on culturally and linguistically diverse (CALD) populations found varying levels of physical activity across studies,^11^ with physical activity participation rates typically declining as population groups aged. Moreover, it was found that CALD populations tend to have lower rates of participation in physical activity such as sports when compared to the general population.^11^ Acculturation, longer time spent in the country and younger age were all associated with increased physical activity in CALD populations. The reasons for low participation were linked to the limited understanding of factors relating to culture and language of CALD populations by the people who were implementing the interventions.^11^ The majority of CALD older adults experience acculturative challenges as they are recent migrants to the host countries and had lesser acculturation time. Building from O’Driscoll and colleagues’ systematic review on physical activity among general CALD populations,^11^ this review was designed to focus on CALD older adults and determine the factors that enhance engagement in physical activity. We have noted that there is limited contemporary evidence consolidated systematically about physical activity programs among CALD older adults. The identified gap has informed our overall review aim and specific questions.

### 1.1 Review aim and questions

This integrative review aimed to explore community‐based physical activity programs undertaken among CALD older adult populations. It specifically sought answers to the following questions:

1. What are the common characteristics of community‐based physical activity programs that were effective for CALD older adults?
2. What culturally appropriate recommendations can be made for physical activity programs or interventions undertaken in CALD older adult populations?

## 2 METHODS

Research of all methodology types was included in this integrative review. We assumed the inclusion of research using diverse methodologies would contribute to a comprehensive understanding of features of effective community‐based physical activity programs for CALD populations. Further, this inclusive approach assumed that exercise and active participation programs targeting CALD older adults may use complex designs. To ensure a rigorous review process, we systematically followed the Whittemore and Knafl^12^ framework for integrative reviews. Key steps in this framework included problem identification, searching the literature, evaluation of data from identified records, analysing data for included studies and presentation of integrated findings.

### 2.1 Problem identification and search strategy

Following the Whittemore and Knafl framework,^12^ problem identification was undertaken by initial scoping of literature for studies and published systematic reviews about the topic of interest. The third author (ID) conducted an initial scoping search to identify the range of current international and local literature available in the field. This search focused on identifying key authors researching CALD populations and checking their publications on this topic. Relevant articles were used to formulate and refine the search terms to guide the search of the designated databases. Next, a systematic search was conducted using MEDLINE, PubMed, Google Scholar, Scopus and CINAHL to locate qualitative and quantitative literature from December 2018 to September 2019. The search terms included CALD, exercise and older adults. These terms were accompanied by terms with the same meaning or that were relevant. For example, CALD was used in conjunction with ‘linguistically diverse’, ‘culturally diverse’ or ‘ethnically diverse’. Other terms such as ‘migrant’, ‘immigrant’, ‘minority’, ‘foreign born’, ‘born abroad’, ‘late life’ or ‘immigrant’ were also used to ensure retrieval of all relevant literature. When searching for physical activity components of CALD research, exercise was truncated as ‘Exerc*’ and used in conjunction with ‘physical activity’, ‘resistance training’, ‘strength training,’ ‘Tai Chi’, ‘Pilates’, ‘aerobic training’, ‘balance training’, ‘group training’ or ‘physical act*’. To ensure we had undertaken a comprehensive search related to our target population, the terms ‘older adults’, ‘elderly’, ‘old adult’, ‘over 65’, ‘senior’, ‘ageing population’, ‘retiree’ or ‘older person’ were used. These search terms were intended to identify a range of relevant literature.

### 2.2 Inclusion criteria

Inclusion criteria for article selection were as follows:

- Published in a peer‐reviewed journal written in English or translated English version;
- Participants were identified in the studies as culturally and linguistically diverse or ‘CALD’;
- Participants were identified as ‘older adults’ or equivalent, based on a chronological age of ≥ 65 years (or justified as an older adult within their cultural context or national definition);
- The intervention had a physical activity or exercise program component undertaken in community‐based settings such as local community centres, organisations or groups.
- The exercise/activity program included a cultural adaptation or culture‐specific design; and
- Culturally specific program design outcomes were formally evaluated.

### 2.3 Exclusion criteria

- Articles that reported participants as indigenous populations (or African American in the USA) were excluded as they are not CALD populations. This methodological decision was based on the definition of CALD and non‐CALD that previous researchers used. In a literature review, O’Driscoll and colleagues^11^ defined CALD as those individuals who were from a particular cultural background and have a distinct native language from the mainstream population in a host country. Indigenous populations, although they have a distinct native language and some may have limited fluency in the mainstream language, were considered non‐CALD, as they were not migrants. We also considered the fact and quoted that ‘African American populations have no linguistic diversity to Anglo‐Americans and have been settled in the USA for approximately 10 generations’^11^ (p.517).
- Programs undertaken in non–community‐based settings such as hospitals and aged care facilities were excluded from this review.

### 2.4 Screening

**Figure 1:**
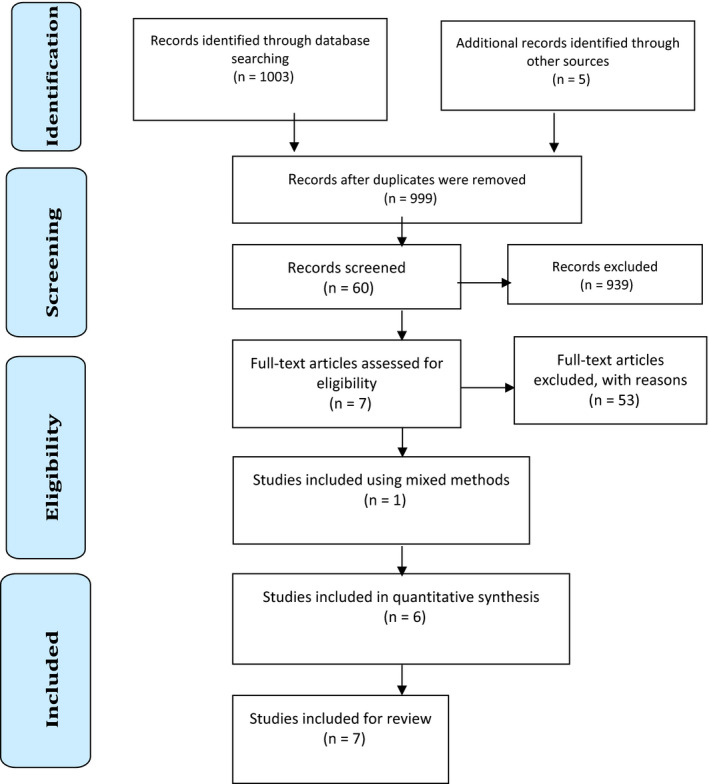
PRISMA 2009 flow diagram detailing identification and selection of included studies^15^

One author (ID) screened the abstracts of identified articles against the inclusion and exclusion criteria. Next, full‐text versions of relevant articles (n = 60) were read and screened against the inclusion and exclusion criteria. In total, 17 articles were identified for potential selection and independently reviewed by two authors (JM and SN) who were external to the literature retrieval process. These authors disagreed on at least one inclusion criterion for five of these articles, which were then screened independently by two other authors (VW and JS‐R). Consensus regarding article selection was reached through discussion. Seven articles that reported results for six studies were selected for this integrative review. Two articles from the same study^13^, ^14^ were included, as each publication reported discreet outcome measures and study results. All selected articles were critically appraised to ensure research rigour (Figure 1).

### 2.5 Critical appraisal

Consensus on the appropriate critical appraisal tools for this integrative review occurred at the beginning of the process. Randomised controlled trials^5^, ^16^ were appraised using the Downs and Black Checklist for Clinical Trial Quality Assessment. ‘Test‐retest’ studies^13^, ^14^, ^17^, ^18^ were appraised using the McMaster University Quantitative Critical Review Form. Finally, the mixed‐methods study^19^ was appraised using the Mixed Methods Appraisal Tool. The initial critical appraisal was completed by one author (ID), a research student who received relevant training on undertaking integrative reviews. The first and second authors (JM and SN) completed an independent critical appraisal process. All appraisal scores were collated and compared. Five discrepancies were considered by the research team, and consensus was reached. Seven articles from six studies were deemed of sufficient quality and included in this integrative review.

### 2.6 Data analysis, abstraction and synthesis

The Whitmore and Knafl^12^ framework was used for data analysis. Data were extracted from full‐text copies of the selected articles, and study findings were grouped. Data reduction was performed to categorise data by topic relevance, and data displays or a schematic diagram was created to check associations and comparisons for commonalities, which in turn formed themes. The synthesis of the results was initially conducted by one author (ID). All authors then brainstormed to evaluate the key characteristics of each study and extract features that may have contributed to the successful outcome of the program.

A preliminary data synthesis was conducted to compare mutual features among the articles, and identify the most common program features. Two independent authors analysed the data, and consensus was reached regarding the cultural adaptation features that contributed to the effectiveness of the reported programs for CALD participants. Conclusions were then drawn subject to verification of data and rechecking against the original studies. Articles with low quality scores from the appraisal were treated with caution in terms of integration and contribution to the main results.

## 3 RESULTS

### 3.1 Data characteristics

In total, we selected seven articles,^5^, ^13^, ^14^, ^15^, ^16^, ^17^, ^18^, ^19^ which reported the results from six studies focused on physical activity or exercise programs for CALD older adults. All of the articles used intervention designs: four used quasi‐experimental, test‐retest designs^13^, ^14^, ^17^, ^18^; two were randomised controlled trials^5^, ^16^; and one used a mixed‐methods design with intervention and qualitative descriptive components.^19^ The CALD populations in the selected articles were Hispanic/Latino,^16^ Korean,^19^ Chinese^13^, ^14^, ^17^ and Turkish.^5^ One study used a multi‐ethnic population with Chinese, Latino and European older immigrants.^18^ The majority of the participants was ≥ 60 years old.^16^, ^17^, ^19^ The oldest reported participant was aged 86 years.^19^ Four of the six studies reported in the selected articles were conducted in the USA,^13^, ^14^, ^17^, ^19^ one was conducted in Canada,^18^ and one was conducted in the Netherlands.^5^ Table 1 presents a summary of articles included in the review.

**Table 1:** Summary of reviewed studies

Article No.	1	2	3	4	5	6a	6b
Authors (year)	Dogra, S., Shah, S., Patel, M., & Tamim, H. (2015)	Lu, Y., DiPierro, M., Chen, L., Chin, R., Fava, M., & Yeung, A. (2014)	Piedra, L. M., Andrade, F. C. D., Hernandez, R., Boughton, S. W., Trejo, L., & Sarkisian, C. A. (2017)	Reijneveld, S. A., Westhoff, M. H., & Hopman‐Rock, M. (2003)	Sin, M. K., Belza, B., LoGerfo, J., & Cunningham, S. (2005)	Taylor‐Piliae, R. E., Haskell, W. L., Stotts, N. A., & Froelicher, E. S (2006a)	Taylor‐Piliae, R. E., Haskell, W. L., Waters, C. M., & Froelicher, E. S. (2006b)
Research design	Test‐retest	Test‐retest	Randomised controlled trial	Randomised controlled trial	Mixed methods	Test‐retest	Same as 6a
Research purpose	To assess the effectiveness of an intervention for improving functional fitness and self‐reported general health among CALD older immigrants with arthritis	To evaluate the feasibility and outcome of a community‐based lifestyle management program for Chinese older immigrants with chronic conditions	To examine the effect of attribution retraining^a^ compared with generic health education on cognitive health over time for CALD older adults enrolled in a low‐cost exercise program	To assess the effect of a short health education and physical exercise program on the health and physical activity of Turkish first‐generation older immigrants	To evaluate the feasibility and effectiveness of a modified exercise program for older Korean immigrants	To examine whether Tai Chi exercise improves performance‐based physical function for older Chinese immigrants with cardiovascular disease risk factors	To examine whether Tai Chi exercise improves psychosocial status for ethnic Chinese with cardiovascular disease risk factors
Sample size	N = 79 F = 71, M = 8	N = 99 F = 57, M = 42	N = 571 F = 441, M = 130	N = 44 (of 92) F = 98, M = 28	N = 13 F = 8, M = 5	N = 39 F = 12, M = 27	Same as 6a
Ages	Aged 50+ mean age = 67.8	Aged 60+	Aged 60+ mean age = 73.12	Aged 55+ (subgroup of all participants aged 45+)	Aged 60+ range 67‐86, mean age = 77	Aged 45+ mean age = 66	Same as 6a
Older CALD immigrant	Chinese (41%) Sth American (23%) European (10%) Caribbean South Asian	Chinese	Hispanic Latino	Turkish	Korean	Chinese	Same as 6a
Residence	Two low‐income neighbourhoods, Toronto	‘Chinatown’ and neighbouring areas, Boston	27 senior centres, Los Angeles	Community dwelling in six cities	One low‐income senior house, Seattle	Community dwelling, San Francisco Bay Area	Same as 6a
Country	Canada	USA	USA	Netherlands	USA	USA	Same as 6a
Physical activity or exercise program component	16‐wk TC program. 1‐hour sessions of Qigong + Yang‐style TC. Participants advised to attend 2 of the 6‐8 weekly group classes offered. 3x occurrences with different cohorts	6‐mth YMCA Health Habits program with access to individual and group exercise facilities, plus required weekly 1‐hr group education sessions on healthy exercise, diet and disease self‐management	EF (modified LFP) 4‐wk core program of 1‐hr exercise class plus 1‐hr/wk group discussion sessions either attribution retraining (treatment) or generic health education (control). Follow‐up at 1yr and 2yrs	8‐wk adapted H&V program (treatment), or 6‐wk existing older adults’ program through welfare services (control)	12‐wk modified LFP, 3x weekly 50‐min group classes	12‐wk TC Yang‐style 24‐posture short form, 3x per week for 60 mins per session, up to 20 person groups	Same as 6a
Cultural‐specific adaptations	Local community centre in locality with high immigrant population. Free of charge. TC master hired for each group. TC master for Chinese‐only program (2nd of 3 cohorts) spoke native language	Community YMCA within ‘Chinatown’ for easy access, nominal 6‐month membership fee charged, or waived for financial hardship, bilingual and bicultural program staff, group education sessions in Chinese language, encouraged social interactions, feedback encouraged	Culturally tailored attribution retraining or health education discussion groups, led by bilingual health educator, low cost	Feasibility for adaptation conducted with 12 Turkish women in 3 focus groups. Program education adapted to older Turks’ culture and knowledge, same‐gender groups, run by Turkish peer educator, using Turkish language, outcome measures translated to Turkish	Bilingual instructor raised in Korea, familiar with Korean language and culture, exercised to Korean 1970s‐80s popular music, satisfaction evaluated in qualitative focus group interview in Korean language	TC deemed culturally appropriate, tailored for Cantonese‐speaking participants, translator provided detailed explanations in all sessions, local community centre with convenient access, setting familiarity	Same as 6a
Cultural adaptation evaluation methods	Program adherence	Program adherence; Continuation rate postintervention; Participant feedback	Program adherence; Diary of activities and problems encountered	Program adherence; Mental well‐being; Participant interviews	Program adherence; Postintervention focus group interview, incl. program appropriateness and satisfaction	Program adherence; Social support changes and piloted measures’ appropriateness and meanings (reported 6b);	Same as 6a
Results	23% of 102 enrolled lost to follow‐up in wk 16. Attendance: average = 1 of 2 recommended sessions per wk; 34% attended ≥ 24 sessions, 31% attended < 8. Statistically significant improvements in: functional fitness LH grip *(P* = .002), CH grip *(P* = .006), Chair stand *(P* < .001), arm curl *(P* < .001), TUG *(P* = .008); and general health (SF‐36) physical component summary *(P* = .001) and subscales: Physical functioning *(P* = .001), Vitality (emotional stability) *(P* = .01), Mental health *(P* = .04).	50% of participants attended ≥ 70% of 1‐hr education sessions, mean attendance 63%. 75% did exercise visits over 6 mths, with 11.6 average visits per month. Statistically significant baseline‐final test mean difference for ↓ body weight *(P*=<0.001), ↓ BMI *(P* < .001), ↓ systolic BP *(P* < .001), ↓ diastolic BP *(P*=<0.001), ↑ reps 30‐s chair stand test *(P* < .001), ↑ one‐leg stance test time *(P* < .001) and ↓ PHQ‐9 *(P* < .01). No significant difference on the WHODAS‐II and UNWG scales. Participant feedback was very positive. >60% continued YMCA membership	80% attendance rate for both groups for 4‐wk core program. 76% of 279 treatment and 71% of 292 control group completed 2‐year follow‐up. Statistically significant ↑ 3MS cognitive functioning scores for both groups baseline‐1yr *(P* = .001); no significant change 1yr‐2yrs. Overall, no significant difference in cognitive function between the treatment and control groups over time.	Difference in change between the treatment and control subgroup participants (age 55+) was strongly positive for improved mental health and statistically significantly *(P = *.01) improved for general mental well‐being. No effect shown for those age 55 + for physical well‐being, physical activity or health knowledge. Compared to the whole treatment group, those age 55 + showed more improved mental health and mental well‐being than their younger counterparts	Program adherence was good with 9 participants attending 80% and 4 attending 60%‐79% of sessions. Health outcomes improved: statistically significantly improved arm strength *(P* < .01) and systolic BP *(P = *.03), lower mean scores on TUG. Participant satisfaction was strong: they felt good and wanted the program to continue, it was fun, and liked the Korean music. Suggested a larger venue where more older Koreans gather such as a seniors’ centre	Program adherence was high 87%. Statistically significant improvements in all performance‐based outcome measures over 12 weeks. Balance improved as measured by functional reach (FR) *(P* = .001), SLS right *(P* = .05) and SLS left *(P* = .001); muscular strength and endurance improved for arm curl *(P* = .001) and chair stand *(P* = .001); and flexibility improved for back scratch *(P* = .001) and CSR *(P* = .001)	Statistically significant improvement in all psychosocial status measures, but not all subscales, over 12 weeks. Perceived stress *(P = *.009) and total mood disturbance *(P = *.008), especially tension‐anxiety *(P = *.002), decreased. Overall social support *(P = *.008), especially from significant others *(P = *.004) and perceived self‐efficacy to i) overcome barriers to Tai Chi exercise (TCSE barriers) *(P = *.001) and to ii) perform Tai Chi continuously for 30 mins (TCSE performance) *(P = *.001) increased
Critical appraisal tool Score	Quantitative studies. McMaster University 12/14	Quantitative studies. McMaster University 13/14	Quantitative studies. McMaster University Downs and Black 19/34 12/14	Quantitative studies. McMaster University Downs and Black 23/34 12/14	Mixed Methods Appraisal Tool—version 2011 3/4 Qualitative 4/4 Quantitative 2/3 mixed method	Quantitative studies. McMaster University 14/14	Quantitative studies. McMaster University Same as 6a

Note

3MS, Modified Mini‐Mental State; BMI, body mass index; BP, Blood pressure; CALD, culturally and linguistically diverse; CH, combined hand; EF, EnhanceFitness; H&V, Healthy and Vital; LFP, Lifetime Fitness Program; LH, left hand; PHQ‐9, Patient Health Questionnaire 9‐item scale (higher score indicates higher levels of depression severity); SF‐36, Short Form‐36; TC, Tai Chi; TUG, Timed Up and Go; UNWG, UN Washington Group disability scale; WHODAS‐II, World Health Organization Disability Assessment Schedule in activities of daily living.

^a^ Attribution retraining aims to raise expectations for ageing by altering participants’ sense of control over personal behaviour and functioning.

### 3.2 Key review findings

All of the reviewed articles that utilised culturally specific interventions were noted to have effective outcomes particularly with program adherence. Five articles reported further evaluation methods, such as preassessment of participants’ acceptance and understanding of the outcome measures,^14^ participant feedback or interviews^5^, ^17^, ^19^ and participants’ diaries or records of any identified problems.^16^ From these, three common culturally appropriate features were identified from the interventions that reported increased program effectiveness for CALD participants. These are (i) occurring at a venue ‘close to home’; (ii) being delivered in participants’ ‘native language’; and (iii) including ‘culturally familiar activities’.

#### 3.2.1 Close to home

The programs reported in the selected studies were strategically designed for delivery in locations with high immigrant populations. Therefore, they were deemed geographically close to home and convenient for CALD participants to attend. In addition, geographically focused recruitment strategies were reported in four articles.^17^, ^18^, ^20^, ^21^ One article^19^ reported the intervention program was delivered in a communal area of the housing facility in which all participants resided. The programs described in the remaining articles were implemented in community or older people's centres that were within (or geographically close to) participants’ home neighbourhoods. All articles reported a continuous participation rate of 60% or over. The highest program participation rate of 87% at 12 weeks, as reported in the studies reviewed was conducted in the closest geographical location or within the housing facility.

#### 3.2.2 Native language instruction

Removing potential communication barriers was an integral feature designed to promote the success of the physical activity or exercise program for CALD participants. All articles described aspects of instruction or conversation in participants’ native languages; however, there were some variations. Bilingual staff or group facilitators^5^, ^16^, ^17^, ^18^, ^19^ or translators^13^, ^14^ who were familiar with participants’ culture were commonly used for program instruction and intervention. Two articles^18^, ^19^ used instructors who were ethnically identical to intervention participants. Lu et al^17^ facilitated their health education program entirely in their participants’ native language, whereas Sin et al^19^ used presession introductions in participants’ native language (Korean) to encourage exercise participation.

#### 3.2.3 Culturally familiar activity

Most of the reviewed interventions embedded culturally familiar activities in the program design. For example, Tai Chi was used as the main intervention for participants who were solely Chinese^13^, ^14^ and for a multi‐ethnic group that was predominantly Chinese.^18^ One program^19^ promoted cultural familiarity for its Korean participants by playing 1970s and 1980s popular Korean music during the exercise program. Those participants reported this intervention was an enjoyable activity. The study by Piedra et al^16^ that included Hispanic/Latino participants used a culturally tailored attribution (behavioural) training intervention. Of significance, the exercise programs that did not use specific, culturally familiar activities (despite including native language instruction) revealed no improvement in physical or health outcomes and had lower participation and attendance rates compared with those that used such activities.^16^, ^18^

All included articles reported using multimodal strategies to recruit participants and retain engagement in the intervention. Such strategies were reported to help build rapport with participants and keep participants engaged throughout the duration and in all aspects of the study. For example, some studies recognised the importance of native language even at the point of evaluation^13^, ^14^ by using the Chinese versions of the outcome measures, including the Cohen Perceived Stress Scale, Profile of Mood States, Multidimensional Scale of Perceived Social Support and the Tai Chi exercise self‐efficacy measure.

## 4 DISCUSSION

This integrative review identified the strategic use of close neighbourhood venues, native language instruction and culturally familiar activities as key features of effective physical activity programs for CALD older adults. These three program design features highlighted the importance of geographic, cultural accessibility, ease of communication and sense of belonging through performing familiar physical leisure activities for health benefits to participants. Across all reported studies, cultural adaptations in the interventions for specific CALD populations were evident to varying degrees, including the choice of exercise themes and activities, outcome measures, recruitment methods, program staff and program review methods. In particular, the use of bilingual staff and facilitators who were culturally and ethnically similar to program participants was reported as an effective method. Older immigrants’ feelings of belonging among co‐ethnic peoples have been reported in other studies of participation.^20^ Designing a culturally meaningful milieu is evident in numerous intervention studies with CALD or indigenous populations that did not meet all of the inclusion criteria for this integrative review.^10^, ^21^, ^22^, ^23^, ^24^, ^25^, ^26^

The included articles reported bespoke intervention programs^13^, ^14^, ^18^ and modified versions of existing community programs^5^, ^16^, ^17^, ^19^ as culturally suitable for enrolling CALD older adults. Similarly, examples of robust bespoke^10^ and modified mainstream^27^ physical activity programs for indigenous populations have been reported in the literature.

Program adherence was the common evaluation method used in the included articles to report program effectiveness for CALD participants. However, adherence is only a proxy measure. Good practice for the evaluation of the effectiveness of cultural adaptations of a program was evident in articles that reported using individual/group interviews to obtain participants’ views on cultural appropriateness and acceptability. The best example was the involvement of participants in a semi‐structured focus group following the intervention program,^19^ which allowed for deep and broad exploration of participants’ views and experiences of the culturally appropriateness of the program features.^18^ The snowballing of ideas that happens in facilitated co‐ethnic group discussions held in participants’ first language may make focus groups an appropriate method to evaluate the effectiveness of culturally specific programs.

The effective program features synthesised from this integrative review were loosely aligned with the Pen‐3 framework, which situates cultural needs as a core value to explain cultural influences on health outcomes.^28^ The Pen‐3 model is built on three distinct components: cultural identification; relationships and expectations; and cultural empowerment. Although none of the articles included in this systemic review referenced the Pen‐3 or any other cultural‐relevant framework, commonalities were evident between the identified features of effective programs and the essential components of the framework. For example, use of ‘cultural identity’, including the person, extended family and neighbourhood, ‘relationships and expectations’ and ‘cultural empowerment’ in the Pen‐3 is conceptually similar to the effectiveness of close‐to‐home venues, native language instruction and culturally familiar activity programs that we found were used to promote program engagement and effectiveness for CALD older adults.

There is limited research involving CALD older adult populations that aims to understand factors that promote their uptake of physical activity or exercise programs at a level that reaches physical activity guidelines for older adults. Importantly, previous research has explored participants’ perceptions of what influenced group exercise participation among CALD populations. For example, Belza et al^21^ found CALD older adult participants expressed the importance of exercise education, building relationships between group participants, culturally tailored exercises and offering low‐ to no‐cost exercise classes at local residential sites.^21^ Walking has also been reported by CALD older adults as an exercise of choice.^26^ Purath, van Son and Corbett^26^ explored Russian‐speaking Slavic immigrants’ views of physical activity; participants described the ability to move as ‘God‐given’ and walking as a ‘gift from God’, and preferred to exercise through performing everyday life activities (eg gardening or housework) rather than joining expensive fitness clubs.

### 4.1 Limitations

As with any review that utilised secondary data, there were limitations in terms of the relevance and generalisability of the findings. This review focused on interventions implemented among CALD populations only and did not explore features of interventions and outcomes with non‐CALD groups. Future research should consider a comparative exploration of program strategies with non‐CALD populations. It is possible that some research related to community‐based physical activity programs for CALD older adults may have not been indexed correctly in electronic databases, and therefore, some relevant studies may have been overlooked in this review.

## 5 CONCLUSIONS

This integrative review sought to identify factors that made community‐based physical activity and exercise programs effective for CALD older adults in their host societies. The synthesised results showed programs that used ‘close‐to‐home’ venues, native language instruction and culturally familiar activities were deemed effective, as evaluated by adherence rates and participants’ subjective experiences. There is a need for studies on physical activity and exercise among CALD older adult populations to evaluate the effectiveness of culturally tailored program features from participants’ perspectives and experiences, rather than relying on proxy measures of effectiveness such as program adherence. The concept of co‐design strategies and interventions with older people is also an emerging methodology that would be useful for optimising CALD groups’ engagement in physical activity and exercises. It is beyond the scope of this integrative review to recommend best practice guidelines. However, the common features of effective programs identified in this review could inform the adaptation of existing community programs to make them culturally appropriate for specific ethnic groups.
